# Study on energy dissipation law of vibration disturbance damaged Zhanjiang Formation structural clay

**DOI:** 10.1371/journal.pone.0325285

**Published:** 2025-06-06

**Authors:** Yanhua Xie, Bin Tang, Shuaiyu Liu, Binghui Zhang

**Affiliations:** 1 Guangxi Key Laboratory of Geomechanics and Geotechnical Engineering, Guilin University of Technology, Guilin, China; 2 School of Energy Engineering and Building Environment, Guilin University of Aerospace Technology, Guilin, China; 3 School of Civil Engineering and Transportation, South China University of Technology, Guangzhou, China; Jazan University College of Engineering, SAUDI ARABIA

## Abstract

This study aims to investigate the energy dissipation laws and underlying mechanisms of damage in Zhanjiang Formation structural clay following vibrational disturbances. Based on the test results of the unconfined compressive strength of the disturbed Zhanjiang Formation structural clay, and in conjunction with theories related to energy dissipation, the analysis revealed that: (1) An increase in the disturbance degree leads to higher compaction strain and compaction dissipation energy, thereby enhancing the ductility of the sample; (2) Although disturbance reduces the strength of the sample, it increases its deformation capacity, indicating that the interplay between strength and deformation affects the level of energy dissipation; (3) The variation in the logarithmic value of the elastic energy dissipation ratio can be categorized into three phases: initial lg*K* > 0; lg*K* < 0 to (lg*K*)_min_; and an increase from (lg*K*)_min_, with (lg*K*)_min_ marking the critical turning point for the transition from the elastic to the plastic phase of the soil. The study also profoundly analyzed the dynamic regulation mechanisms of vibrational disturbance energy from two perspectives: dynamic regulation and structural change, as well as energy dissipation and damage evolution. High-frequency vibrations expedite the damage process, and post-damage energy dissipation involves friction, microcrack propagation, and plastic deformation, ultimately leading to the collapse of the soil structure. This study not only enhances the understanding of the complex mechanical behavior of such soils but also paves the way for innovative applications and theoretical advances in soil mechanics.

## Introduction

The mechanical properties of soil (strength and deformation) are key indicators in engineering construction and have long been a focal point of attention in both the engineering and academic communities. Clay with pronounced structural features commonly exhibits complex and variable engineering properties that significantly affect the safety and stability of engineering projects. Of these, the Zhanjiang Formation structural clay is particularly well represented. This type of soil possesses undesirable physical properties, such as high-water content, elevated porosity ratio, and extreme sensitivity, yet its undisturbed state exhibits relatively strong mechanical properties [1]. This presents an anomalous combination of poor physical characteristics and favorable mechanical property indicators [2], posing challenges for engineering construction. In particular, the mechanical response and energy dissipation mechanisms of the Zhanjiang Formation structural clays become particularly complex upon disturbance, requiring further in-depth investigations.

In response to this challenge, both the academic and engineering communities have conducted extensive research aimed at uncovering the intrinsic mechanisms and proposing effective engineering countermeasures. On the one hand, through techniques such as microstructure analysis [3], electron microscopy scanning [4], and X-ray diffraction [5], the relationship between the microstructure and macroscopic mechanical behavior of soil is explored in depth, providing a scientific basis for understanding its complex properties. On the other hand, a large number of laboratory experiments and field tests have been carried out to simulate the soil response under different working conditions [6,7]. Furthermore, considering the widespread distribution of the Zhanjiang Formation structural clay and the diversity of engineering needs [8], establishing constitutive models suitable for such soils has become a research focus [9–11]. These models are required to accurately describe the stress-strain relationship of soil under different stress pathways and water content conditions, and to take into account the effects of structural damage on mechanical properties, providing more precise predictive tools for numerical simulations and engineering design. The aforementioned studies have laid a solid foundation for a deeper understanding of the complex mechanical behavior of the Zhanjiang Formation structural clay.

It is essential to note that the essence of soil damage lies in the process of energy transformation and dissipation [12]. When subjected to external disturbances such as construction activities, the expansion of microscopic defects, the sliding between particles, and changes in the pore structure within the soil result in significant alterations to its mechanical properties. Therefore, studying the mechanical response and damage mechanisms of Zhanjiang Formation structural clay from an energy perspective provides a fresh perspective to understand the evolution of its macroscopic mechanical behavior. In particular, the analysis of the distribution of dissipation energy and elastic energy in the total absorbed energy is crucial to reveal the intrinsic relationship between the accumulation of structural damage and the degradation of mechanical properties.

This study is based on the energy method to conduct unconfined compressive strength tests on damaged Zhanjiang Formation structural clay after vibration disturbance. The stress-strain relation of the damaged structural clay after vibration disturbance is analyzed, the location of the compaction point of the sample is determined, and the energy evolution and distribution rules are explored. On this basis, the energy evolution mechanism is revealed, which aims to provide a reference for the evaluation of degradation and stability control of damaged structural clays caused by vibration disturbance.

## Test soil and analysis

### Test soil

The soil samples were sourced from Donghai Island, Zhanjiang City, Guangdong Province, China. The primary minerals in the soil are quartz and plagioclase, which account for 50.4%, while the secondary clay minerals are dominated by kaolinite and illite, which account for 49.6%. According to the Chinese national standard “Standard for Geotechnical Test Methods”(GB/T50123-2019) [13], various physical indices of the Zhanjiang Formation structural clay were tested, and the specific indices are presented in Table 1.

**Table 1:** Basic physical properties of Zhanjiang Formation structural clay [14].

Water content (w/% )	Natural density ρ ($g/cm^{3}$)	Dry density ρ ($g/cm^{3}$)	Specific gravity Gs	Plasticity index $I_{P}$	Liquid index $I_{L}$	Void ratio e	Sensitivity Sr
47.22	1.74	1.18	2.72	25.83	0.81	1.29	4.01

### Preparation of vibration disturbance damage samples

To better investigate the stress behavior of damaged structural clay, and in accordance with the current national standard “Standard for allowable vibration of building engineering” (GB/50868–2013) of China [15], which stipulates that the allowable vibration frequency at the foundation should be less than 100 Hz when employing hammering and vibrating methods for pile driving, as well as the vibration compaction method for foundation treatment. Based on the homogenization of the data, vibration frequencies of 20, 35, and 50 Hz were chosen, and vibration durations were set to 30, 60, and 90 mins. Test samples were prepared using an electro-frequency modulated vibrator provided by Hebei Tengfei Test Instrument Co., Ltd., China. The vertical vibrations were set to an amplitude of 0.6 mm. Table 2 shows the selected experimental conditions for various scenarios.

**Table 2:** Parameters of vibration frequency and vibration duration.

Duration Frequency	0min	30min	60min	90min
**0hz**	A1	–	–	–
**20hz**	–	B1	C1	D1
**35hz**	–	B2	C2	D2
**50hz**	–	B3	C3	D3

### Unconfined compressive strength test and analysis

**Fig 1:**
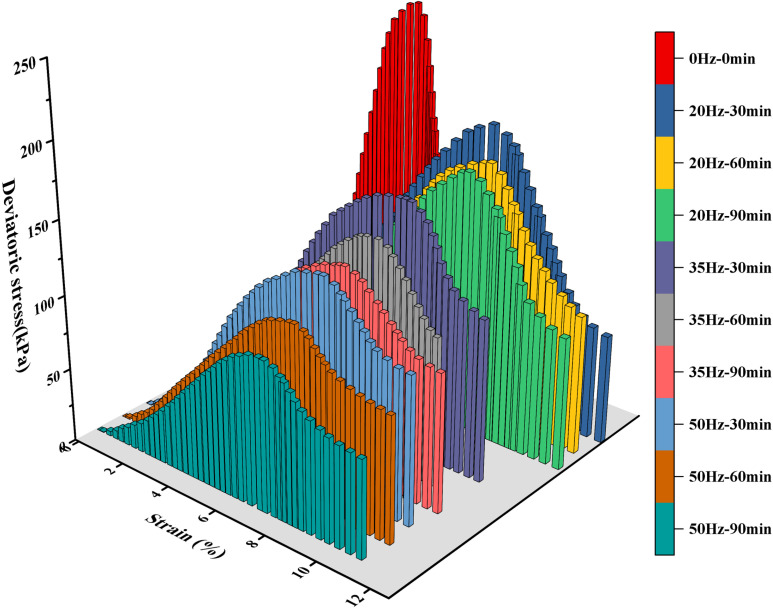
Stress-strain curves under different conditions. Following the vibration disturbance, the stress-strain curve of the sample exhibits strain-softening behavior. The peak deviatoric stress is taken to be the unconfined compressive strength of the sample.

The unconfined compressive strength test is one of the classic mechanical tests in the field of soil mechanics, and a universal test machine was utilized in this experiment. The loading rate was set at 1 mm/min until the samples failed or the axial strain reached 20%. The determination of the test results was based on the test value of a single sample (if the deviation exceeded 10%, the results of the preparatory sample were used). Fig 1 shows the stress-strain curves of the samples under different test conditions. As depicted in Fig 1, the stress-strain curve of the damaged Zhanjiang Formation structural clay following vibration disturbance is of the strain-softening behavior, which can roughly be divided into three distinct stages: firstly, the ascending stage, where the axial stress increased rapidly with the escalated in axial strain, slowing down as it approached the peak; secondly, the descending stage, where the axial stress dropped from the peak to the residual strength and the soil structure was destroyed; and finally, the steady stage, where the axial stress decreased slowly and the sample maintained a certain residual strength and underwent significant plastic deformation. The undisturbed soil, after being disturbed by 20 Hz vibration, showed structural damage, a decrease in axial bearing capacity, a reduction in peak strength, loosening of soil texture, and an increase in axial strain at peak strength. After 60 minutes of vibration, the soil stress-strain curve showed no significant shifts, indicating that extending the vibration duration beyond 60 minutes at 20 Hz did not result in further damage to the soil structure. However, when increasing the vibration frequency to 35 Hz and 50 Hz, the overall stress-strain curves of the soil samples shifted downward with increasing vibration duration, and both the peak and residual strengths decreased.

Fig 1 shows the stress-strain curve, where the peak corresponds to the maximum deviatoric stress, which is the unconfined compressive strength. The test results for the unconfined compressive strength of the specimens under different conditions are given in Table 3.

**Table 3:** Unconfined compressive strength of disturbed samples [14].

Vibration duration/min	Vibration frequency/Hz			
	0	20	35	50
**0**	241.94	–	–	–
**30**	–	187.57	173.24	174.35
**60**	–	166.79	148.15	137.19
**90**	–	139.89	116.89	101.94

### Disturbance degree

Numerous scholars at home and abroad [16–18] have conducted in-depth studies on the definition and evaluation of soil disturbance degree after sampling. Based on changes in pore water pressure, e-lop curves, and alterations in soil strength pre- and post-sampling, they have proposed methods to determine the disturbance degree of soil. In this study, the reduced proportion of unconfined compressive strength was used to define the disturbance degree, which is calculated as follows.


$$RD_{q}=\frac{q_{u}-{q_{u}}^{\prime }}{q_{u}}$$ (1)

Here: *RD*_*q*_ represents the disturbance degree defined by soil strength characteristics, *q*_*u*_ is the unconfined compressive strength of the undisturbed soil, and *q*_*u*_*’* is the unconfined compressive strength of the disturbed soil. The closer the *RD*_*q*_ value approaches 1, the greater the disturbance degree of the soil sample.

Here, *RD*_*q*_ denotes the disturbance degree defined by soil strength properties. Specifically, *q*_*u*_ represents the unconfined compressive strength of the undisturbed soil, while *q*_*u*_*’* signifies the unconfined compressive strength of the disturbed soil. The closer the *RD*_*q*_ value is to 1, the higher the disturbance degree of the soil sample.

The disturbance degree was quantitatively calculated using Equation (1), as detailed in Table 4.

**Table 4:** Disturbance degree (*RD*_*q*_) [1].

Vibration duration/min	Vibration frequency/Hz			
	0	20	35	50
**0**	0	–	–	–
**30**	–	0.22	0.31	0.42
**60**	–	0.28	0.39	0.52
**90**	–	0.28	0.43	0.58

*RD*_*q*_ is the disturbance degree defined in terms of unconfined compressive strength.

### Energy relationship during the damage process

The research methods of damage theory are primarily divided into two categories: energy damage theory and geometric damage theory. Energy damage theory, established by J. Lemaitre et al. [19], is based on continuum mechanics and thermodynamics, and regards the damage process as an irreversible process of energy conversion. In this framework, the mechanical behavior of the continuous medium is equivalent to a thermodynamic process, and the damage is essentially the dissipation of energy.

Given the complexity of soil structure and the diversity of damage forms, it is particularly challenging to delve into the patterns of damage changes at a microscopic level, and at times, it may even be unachievable. However, the common characteristic of various types of damage is that they are irreversible processes that dissipate energy, ultimately leading to the degradation of the macroscopic mechanical properties of the material.

**Fig 2:**
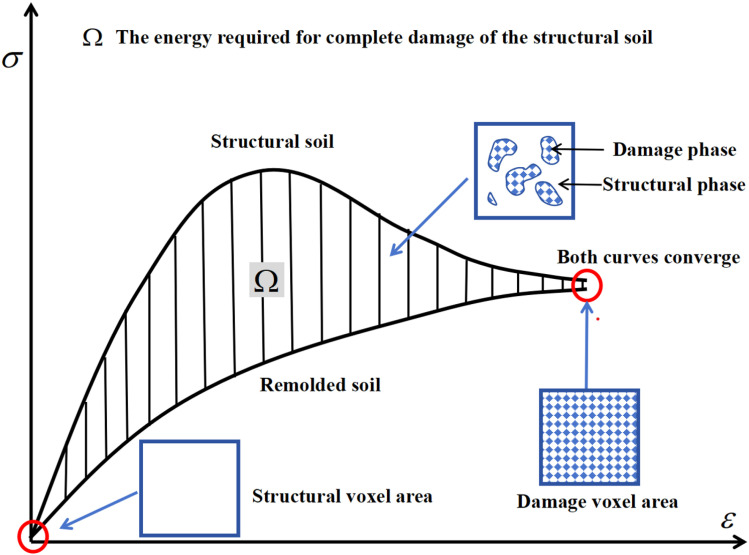
Typical stress-strain curve for soil. Under the action of external forces, structural soil transitions to remolded soil, and the structural phase shifts to the damage phase.

Under stress conditions, remodeled soil has completely lost its original structure, and its stress shows no significant peak, exhibiting a state of complete damage. In contrast, when structural soil is subjected to external loads, the structural phase gradually transitions to the damage phase, resulting in strain softening. Once the structural phase is fully damaged, the stress-strain curves of both converge [15], as illustrated in Fig 2. The shaded *Ω* area in the figure represents the energy dissipated by the complete damage to the structural phase, which is the energy required for complete damage the structural soil. Damage dissipation energy refers to the surface energy needed for the emergence and expansion of internal micro-defects (such as cracks and pores) under external forces until they coalesce [16]. From the perspective of irreversible thermodynamics, the damage to structured soil is an irreversible thermodynamic process, where energy dissipation is a crucial intrinsic factor determining damage development. When the strain energy released by the structural phase exceeds the energy required for the phase transition to the damage phase, unstable damage propagation occurs. This results in a reduction in the area of the structural phase and an increase in the damage phase. In this process, the work done by external loads is primarily converted into the elastic energy of the structural phase and the dissipation energy during the phase transition.

### Energy balance relationship

**Fig 3:**
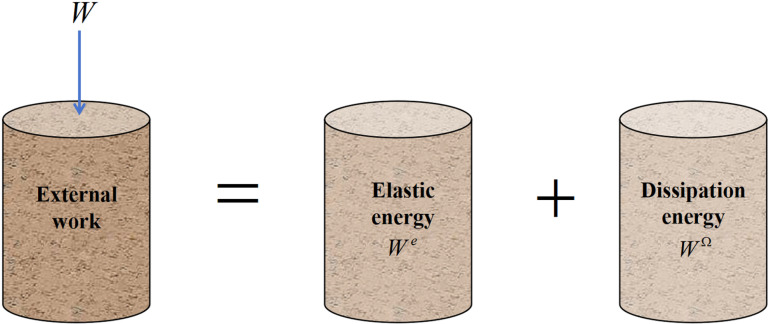
Energy balance relationship of structural soil. The total input energy from external work is converted into elastic energy and dissipation energy.


According to the laws of thermodynamics, energy conversion is the essential characteristic of the physical change process in materials, and material failure is a state instability phenomenon driven by energy [16]. Under mechanical loading, assuming that the soil is disturbed in a closed system without heat exchange with the external environment, the total input energy due to external work will be converted into the internal elastic energy and dissipation energy of the material, as illustrated in Fig 3. Dissipation energy refers to the irreversible energy resulting from internal structural damage of the material, derived from the first law of thermodynamics:

$$W=W^{e}+W^{\Omega }$$ (2)

Where *W* is the work done by the external force, *W*^*e*^ is the elastic energy stored structurally, and *W*^*Ω*^ is the dissipation energy.

**Fig 4:**
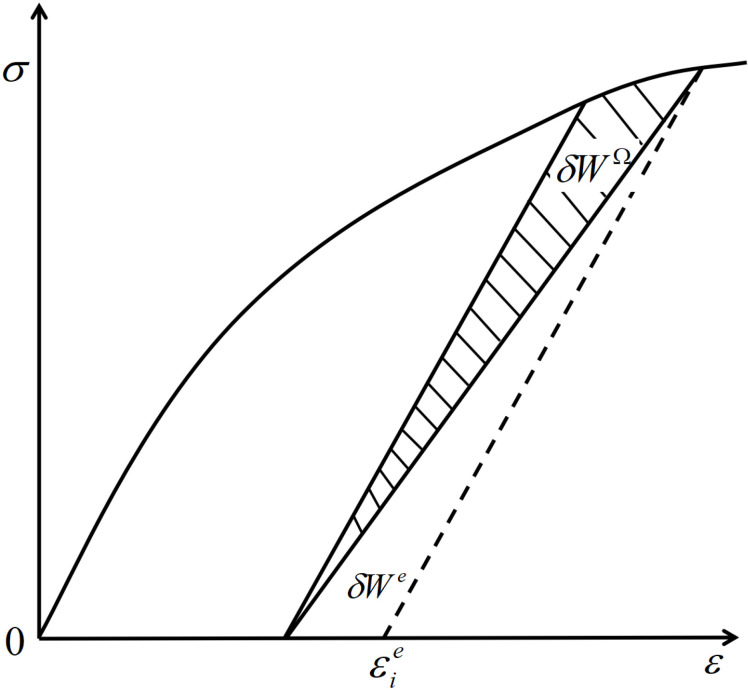
Work and dissipation analysis. During the process of strain δε, the external force energy *δW *= *σδε = δW*^*e *^+ *δW*^*Ω*^.

Damage dissipation energy *Ω* refers to the surface energy required for the formation, development, and penetration of micro-defects such as cracks and voids within materials under external loading. As depicted in Fig 4, during the strain *δε* process, the energy *δW *= *σδε* per unit mass exerted on the voxel by external forces is dissipated and stored. The work of the external force is converted into two components: the first is the recoverable elastic energy *δW*^*e*^, and the second is the irrecoverable dissipation energy *δW*^*Ω*^.

The total strain energy absorbed by the soil can be obtained by integrating the stress-strain curve:


$$W=\int \sigma_{i}d\varepsilon_{i}$$ (3)

Combining Fig 3, it can be concluded:


$$W^{e}=\frac{1}{2}\varepsilon_{i}^{e}\sigma_{i}$$ (4)

To estimate the elastic modulus *E*_*d*_ (Equation 5) [17], the following assumptions are necessary: Assumption 1, the stress-strain relationship of the soil within the selected strain range (i.e., 60% to 40% of *σ*) is approximately linear. It should be noted that a wider strain range may include more nonlinear behaviors, potentially leading to an underestimation of the elastic modulus. Conversely, a narrower strain range may result in insufficient data points, thereby causing significant errors. Assumption 2, the properties of the soil material remain uniform during the test, implying that factors such as internal defects and uneven particle size distribution are not considered to influence the elastic modulus.


$$E_{d}=\frac{\sigma_{60\;\%}-\sigma_{40\;\%}}{\varepsilon_{60\;\%}-\varepsilon_{40\;\%}}$$ (5)

In the formula, *E*_*d*_ denotes the elastic modulus of the disturbed soil, measured in kPa; *σ*_*60%*_ and *σ*_*40%*_ correspond to the stress at 60% and 40% of the peak stress, respectively, in kPa, while *ε*_60%_ and *ε*_60%_ represent the strain at 60% and 40% of the peak strain, respectively. From Equations (3) and (4), it can be deduced that.


$$W^{e}=\frac{\sigma_{i}^{2}}{2E_{d}}=\frac{\sigma_{i}^{2}(\varepsilon_{60\;\%}-\varepsilon_{40\;\%})}{2(\sigma_{60\;\%}-\sigma_{40\;\%})}$$ (6)

From Equations (2), (3), and (6), it can be deduced that.


$$W^{\Omega }=W-W^{e}=\int \sigma_{i}d\varepsilon_{i}-\frac{\sigma_{i}^{2}(\varepsilon_{60\;\%}-\varepsilon_{40\;\%})}{2(\sigma_{60\;\%}-\sigma_{40\;\%})}$$ (7)

### Energy evolution law

The failure process of soil is essentially the result of the continuous evolution of its internal elastic energy and dissipation energy. From an energy perspective, energy directly reflects the combined effects of force and deformation (displacement). Therefore, analyzing the failure process of disturbed soil from an energy perspective provides a more intuitive understanding.

**Fig 5:**
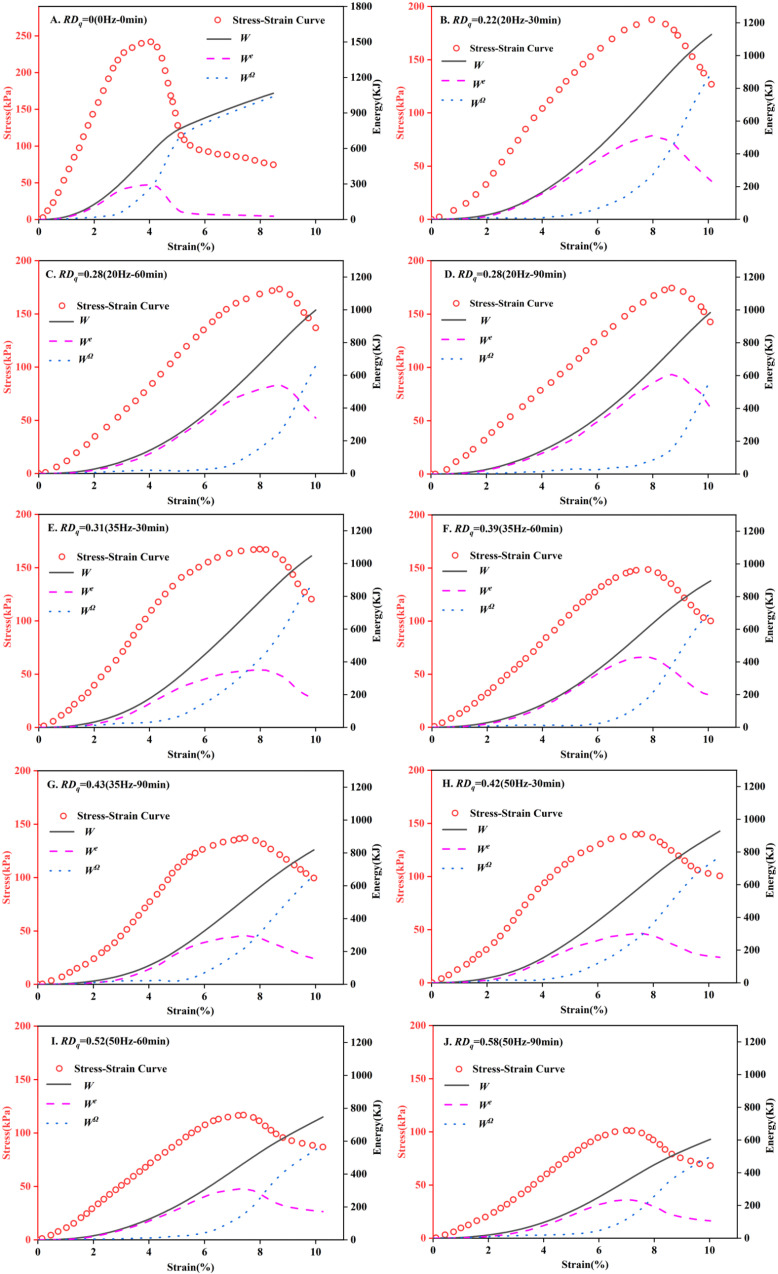
Energy evolution laws of clay with varying disturbance degrees under unconfined compressive conditions. In the figure, *W* represents the total energy input from external forces, *W*^*e*^ denotes the elastic energy stored within the soil, and *W*^*Ω*^ indicates the dissipation energy. These quantities satisfy the relationship *W* = *W*^*e*^ + *W*^*Ω*^. The total energy *W* gradually increases with strain. Both the elastic energy *W*^*e*^ and the stress initially increase but subsequently decrease with strain. The dissipation energy *W*^*Ω*^ can be divided into two phases: a slow initial growth period followed by a rapid growth stage.

Using formulas (3), (6), and (7), the evolution processes of the total energy *W*, elastic energy *W*^*e*^, dissipation energy *W*^*Ω*^, and stress of Zhanjiang Formation structural clay with different disturbance damage degrees were calculated. Fig 5 illustrates the energy evolution laws of Zhanjiang Formation structural clay under unconfined compression conditions at varying disturbance degrees. As shown in the figure, regardless of the disturbance degree, the total energy *W* absorbed increases gradually with strain. Both elastic energy *W*^*e*^ and stress initially increase and then decrease with strain. The change in dissipation energy *W*^*Ω*^ follows two stages: a slow growth phase followed by a rapid growth phase.

For samples with identical disturbance degrees, the initial loading phase corresponds to a compaction stage characterized by adjustments and closures in pore structure. During this stage, the increase in dissipation energy is primarily related to the initial closure of pores, displaying a gradual upward trend. As the sample compacts, its elastic energy also accumulates slowly. Upon entering the elastic stage, where pores are nearly closed, the dissipation energy remains relatively stable. At this juncture, the total energy absorbed is primarily stored in the soil as elastic energy.

### Location of compaction point

**Fig 6:**
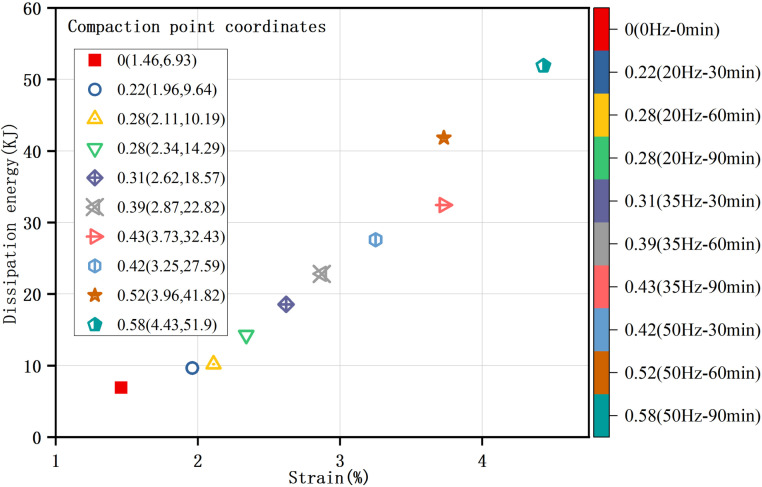
Locations of compaction points of clay with varying disturbance degrees. Based on the variations in dissipation energy during the loading process, the compaction point is defined as the location at which the pores of the sample are completely closed, and the elastic energy begins to accumulate. The compaction point's location of the sample is then determined.

**Fig 7:**
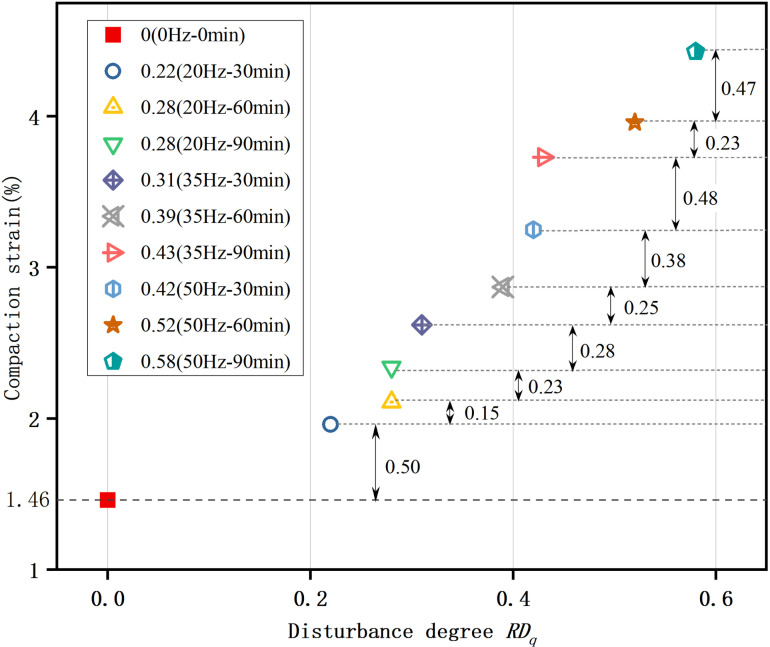
Relationship between disturbance degree and strain at the compaction point. As the disturbance degree increases, there is a corresponding rise in compaction strain, indicating a continual enhancement of the sample's ductility.

**Fig 8:**
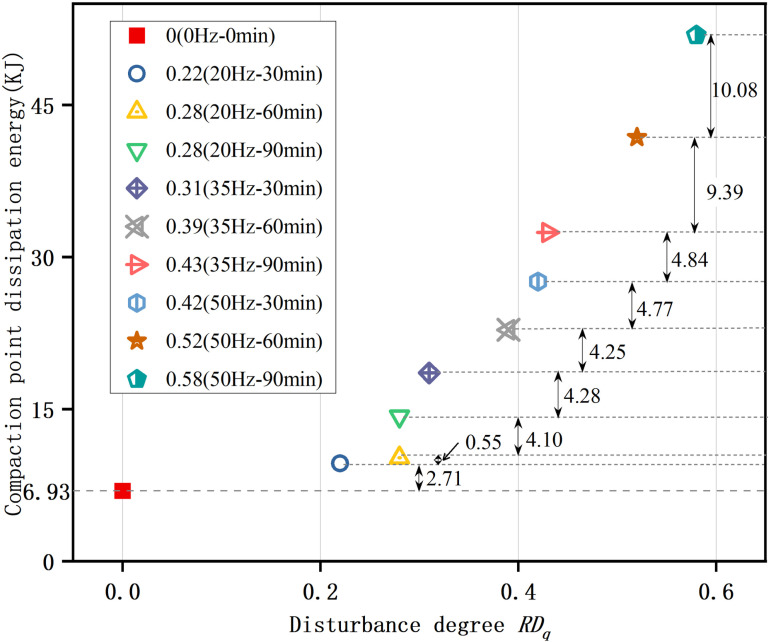
Relationship between disturbance degree and dissipation energy at the compaction point. As the disturbance degree increases, the dissipation energy at the compaction point escalates.

Based on the analysis of the dissipation energy variations depicted in Fig 5, the compaction point is defined as the location at which the sample's pores are completely closed and the accumulation of elastic energy begins. Consequently, Fig 6 is obtained, showing the specific locations of the compaction points, which vary for samples with different disturbance degrees. As demonstrated in Fig 7, an increase in the disturbance degree corresponds to a rise in compaction strain, indicating that the ductility of the sample is continuously enhancing as the disturbance degree increases. Further illustrated by Fig 8, the disturbance degree also contributes to an increase in compaction dissipation energy, which means that samples with higher disturbance degrees contain more pores, necessitating greater dissipation energy to achieve a compact, dense state.

### Energy distribution law

**Fig 9:**
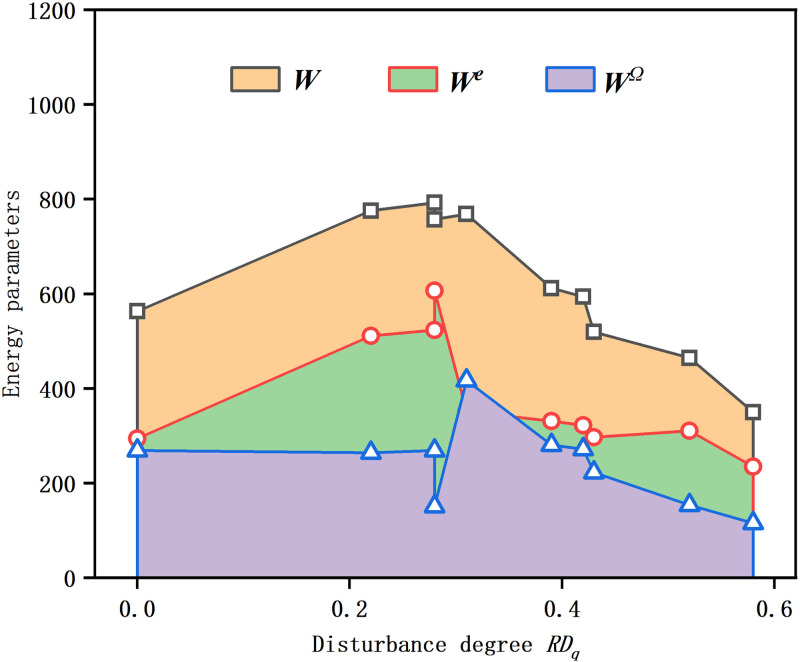
Variations of energy parameters at peak stress of samples with different disturbance degrees. The energy parameters depicted in the figure are as follows: *W* represents the total energy, *W*^*e*^ denotes the elastic energy stored in the soil, *W*^*Ω*^ signifies the dissipation energy, and *W* = *W*^*e *^+ *W*^*Ω*^.

Fig 9 shows the energy distribution corresponding to the peak stress of unconfined compression for samples with different disturbance degrees. From the figure, it can be observed that the change in energy parameters is divided by disturbance degrees of 0.28 and 0.31, showing three distinct trends. Within the range of disturbance degrees from 0 to 0.28, as the disturbance degree increases, the total energy, elastic energy, and dissipation energy all show an upward trend, with specific increases of 40.71%, 77.80%, and 0.08%, respectively. However, for disturbance degrees between 0.31 and 0.58, these energy parameters all decrease, by 54.47%, 33.23%, and 72.36%, respectively. Particularly, in the narrow range from 0.28 to 0.31, the trends of elastic energy and dissipation energy are opposite; the former decreases while the latter increases.

For the Zhanjiang Formation structural clay, although its strength is weakened after disturbance, the increasing trend of dissipation energy at the compaction point indicates an increase in its deformation capability. Since energy is the result of the combined effect of strength and deformation, the overall trend shows an increase in energy up to a disturbance degree of 0.28. This finding is consistent with the conclusions of GAO et al. [20], indicating that disturbance does not necessarily lead to a decrease in energy, but is instead influenced by the combined effect of strength and deformation.

In literature [21–23], the ratio of dissipation energy to elastic energy is defined as the elastic energy dissipation ratio, which is used to reveal the proportional relationship between energy storage and energy dissipation in rock samples during the entire process from loading deformation to failure. Thus, the concept of the elastic energy dissipation ratio *K* is adopted in this study to reflect the correlation between energy storage and energy dissipation in clay.


$$K=\frac{W^{\Omega }}{W^{e}}$$ (8)

In the formula, *W*^*e*^ represents the elastic energy, and *W*^*Ω*^ represents the dissipation energy.

**Fig 10:**
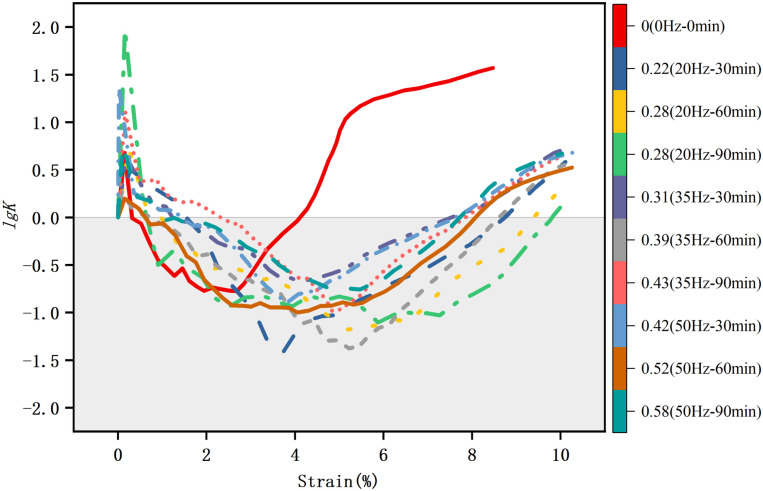
Variation of elastic energy dissipation ratio. The variation in logarithmic value of the elastic energy dissipation ratio can be divided into three stages: initial lg*K *> 0, lg*K* < 0 to (lg*K*)_min_, and rising from (lg*K*)_min_, where (lg*K*)_min_ is considered the crucial turning point from the elastic to the plastic phase of the soil.

The elastic energy dissipation ratio *K*, as a crucial parameter reflecting the proportional relationship between the energy storage and dissipation in soil, can effectively characterize the energy absorption and release capacity of the soil under stress. During the process of the soil approaching failure, its energy storage capacity decreases significantly, while the dissipation energy rapidly increases, leading to a reduction in the *K* value. This process reflects the transition of the soil from elastic to plastic and eventually to the failure stage. Variations in the value of *K* can be regarded as a representation of the internal damage accumulation in the soil. Given the sensitivity and regular changes exhibited by the elastic energy dissipation ratio *K* during the energy transformation process of the soil, it is of great significance to use its logarithmic value, lg*K*, as a key indicator of the critical turning point. Using lg*K* not only expands the range of *K* value variation but also enables it to sensitively reflect subtle differences in the soil properties under different samples or conditions, especially when the *K* values themselves are relatively small or exhibit insignificant differences. Fig 10 shows the variation of the logarithm of the elastic energy dissipation ratio *K* for different disturbed samples. The soil exhibits different characteristics at different logarithmic stages of the elastic energy dissipation ratio *K*: (1) In the initial stage of lg*K *> 0, it corresponds to the pore closure process in the soil. At this time, the pores between the aggregates gradually decrease, the compactness of the soil gradually increases, and the stability continuously improves. (2) When lg*K* < 0 to (lg*K*)_min_, the soil is in the elastic energy storage stage, and the total energy it absorbs is mainly stored in the form of elastic energy. In this stage, the soil can return to its original shape without permanent deformation after being subjected to external forces. The proportion of elastic energy reaches its peak corresponding to the (lg*K*)_min_, indicating that the soil is in the most stable state, which can be considered as the point for the transition from the elastic stage to the plastic stage of the soil. (3) From (lg*K*)_min_ to the failure stage, lg*K* commences to increase from the minimum value, signifying the transition of the soil from the elastic stage to the plastic stage and even failure; when lg*K* > 0 once again, it implies that the soil is approaching or has already failed. Therefore, in terms of the characterization of sample states by lg*K*, from the initial stage where lg*K* > 0, to the elastic energy storage stage where lg*K* < 0 until (lg*K*)_min_, and further from (lg*K*)_min_ to the failure stage, (lg*K*)_min_ can be considered as the critical turning point for the transition from the elastic to the plastic stage of the soil.

Table 5 shows the stress values corresponding to the (lg*K*)_min_ of samples with different disturbance degrees. The data indicate that as the disturbance degree increases, the stress value corresponding to (lg*K*)_min_ decreases continuously, and the proportion of stress relative to the peak strength also diminishes. This suggests that the soil has undergone significant plastic deformation before reaching its ultimate bearing capacity. The increase in this deformation capacity, that is, the enhancement of ductility, enables the soil to absorb more energy under external loads. Therefore, the higher the disturbance degree, the more pronounced the ductility of the soil.

**Table 5:** Critical turning point indicator.

Disturbance degree	$(\mathrm{lg}K{)}_{\min}$	$\sigma_{{\left(\mathrm{lg}K\right)}_{\min}}/kPa$	σ/kPa	Proportion
**0(0 Hz-0 min)**	−0.77	205.93	241.94	85.12%
**0.22(20 Hz-30 min)**	−1.43	145.31	187.57	77.47%
**0.28(20 Hz-60 min)**	−1.18	131.29	173.24	75.79%
**0.28(20 Hz-90 min)**	−1.10	133.68	174.35	76.67%
**0.31(35 Hz-30 min)**	−0.67	128	167.38	76.47%
**0.39(35 Hz-60 min)**	−1.38	109.38	148.56	73.63%
**0.43(35 Hz-90 min)**	−0.98	98.12	137.09	71.57%
**0.42(50 Hz-30 min)**	−0.89	102.3	139.94	73.10%
**0.52(50 Hz-60 min)**	−1.00	73.05	116.72	62.59%
**0.58(50 Hz-90 min)**	−0.76	62.13	101.39	61.28%

The (lg*K*)_min_ refers to the minimum logarithmic value of the elastic energy dissipation ratio *K* of the sample. The *σ*_(lg*K*)min_ represents the stress value corresponding to the minimum logarithmic value of the elastic energy dissipation ratio *K* of the sample. The *σ* denotes the unconfined compressive strength of the sample.

**Fig 11:**
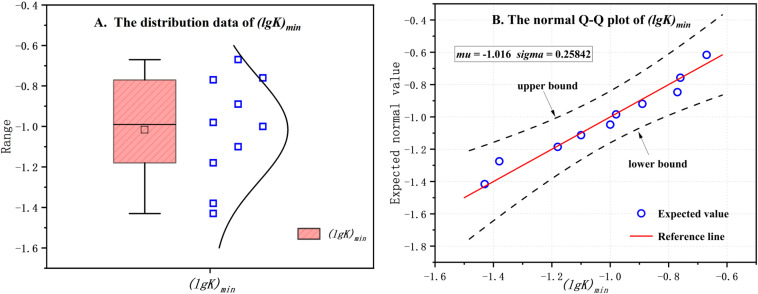
The (lg*K*)_min_ threshold (11(A) (lg*K*)_min_ values distribution, 11(B) The normal Q-Q plot of (lg*K*)_min_). In 11(A), the □ symbol represents the distribution of (lg*K*)_min_ values across samples with varying disturbance degrees. Assuming that (lg*K*)_min_ adheres to a normal distribution, the analysis was performed using a normal Q-Q plot of (lg*K*)_min_, as shown in 11(B). This plot illustrates the alignment of the (lg*K*)_min_ distribution with the theoretical normal distribution.

The (lg*K*)_min_ can be considered as the critical turning point for the transition from the elastic to the plastic stage of the soil. Identifying the threshold range is significant for assessing the mechanical properties of the soil and predicting its state. Through the analysis of (lg*K*)_min_ data, it was hypothesized that (lg*K*)_min_ adheres to a normal distribution. The Quantile-Quantile (Q-Q) plot analysis of (lg*K*)_min_ confirmed its alignment with the theoretical normal distribution, characterized by a mean mu(*μ*) of −1.016 and a standard deviation sigma(*σ*) of 0.25842, as shown in Fig 11. This indicates a relatively narrow range of data fluctuation, with most (lg*K*)_min_ values clustering tightly around the mean of −1.016. Consequently, the threshold range for the critical point (lg*K*)_min_, delineating the transition from the elastic to the plastic stage, has been more precisely defined.

**Fig 12:**
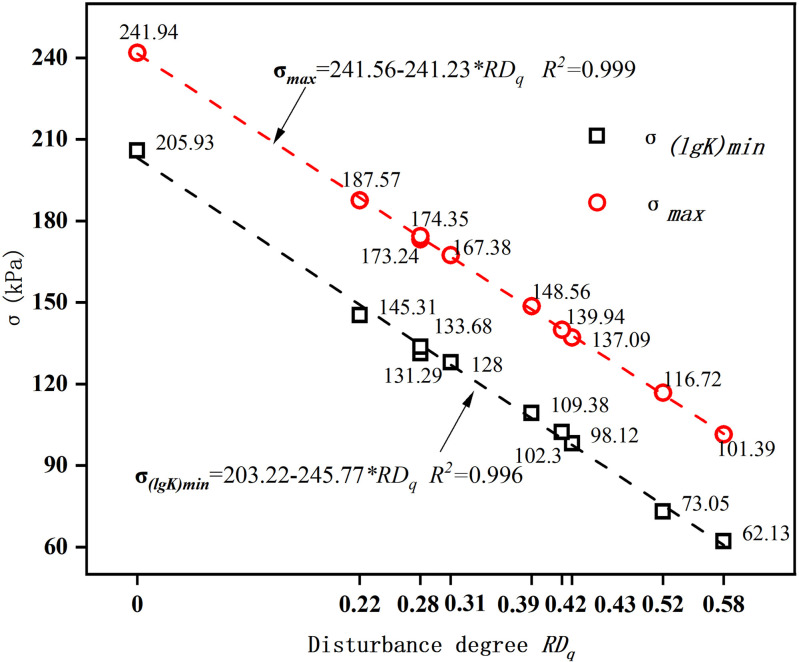
Relationship between disturbance degree and stress indicators before failure of the disturbed samples. Both *σ*_(lg*K*)min_ and *σ* max demonstrate a linear decline as the disturbance degree increases, indicating that higher disturbance levels correspond to a gradual reduction in these stress indicators.

Through linear regression analysis, a linear relationship was established between the disturbance degree and the stress indicator before failure of the disturbed sample, as depicted in Fig 12. This relationship provides an empirical model for the prediction of the soil strength across varying disturbance degrees.

### Analysis of energy dissipation mechanism

After vibration disturbance, the analysis of the energy evolution mechanism of structural clay that incurs further damage is key to understanding the dynamic response characteristics of soil. Structural clay, due to its unique particle arrangement, pore structure, and cementation, undergoes a more complex process of internal energy conversion and dissipation when subjected to external vibratory loads compared to non-structural or weakly structural soils. Therefore, it is necessary to analyze its energy evolution mechanism from the following two aspects.

### Dynamic regulation and structural change

Vibration disturbance, as the primary form of external energy input, is mainly dynamically regulated by precisely controlling the vibration duration and frequency of the disturbance source. Both factors significantly influence the energy dissipation mechanisms of Zhanjiang Formation structural clay. Specifically, short-term high-frequency disturbances may rapidly accumulate pore water pressure within the soil, prompting redistribution of interparticle contact stress, thereby increasing instantaneous deformation and energy dissipation. Conversely, long-term low-frequency disturbances allow sufficient time for internal structural adjustments, such as particle rearrangement and pore compression, which facilitate a more gradual energy dissipation and reduce the risk of sudden failure. Additionally, high-frequency vibrations can exacerbate friction and collisions between soil particles, fostering the formation of microcracks and accelerating cumulative damage. In comparison, low-frequency disturbances predominantly induce overall soil deformation, typically associated with lower energy dissipation but potentially leading to structural degradation under prolonged exposure.

**Fig 13:**
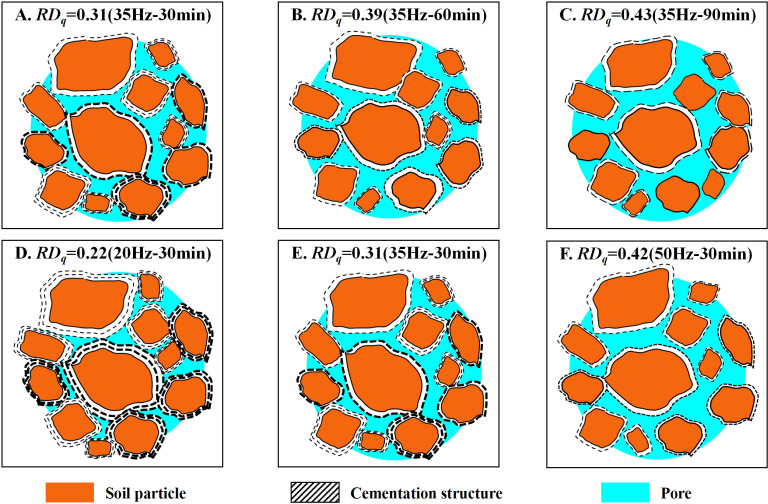
Microstructural Changes. Dynamic regulation is achieved by controlling the vibration duration and frequency of the disturbance source. In Fig 13(A)–13(C), as the duration increases, the collisions and friction between particles intensify, resulting in the formation and expansion of microcracks within the soil. In Fig. 13(D)–13(F), high-frequency vibrations exacerbate the collisions and friction between particles, promoting the rapid expansion of microcracks within the soil and swiftly transitioning into the macroscopic damage phase.

The dynamic regulation effects of vibration duration were analyzed (as shown in Fig 13(A)–13(C)). With prolonged duration, collisions and friction between particles intensified, resulting in the initiation and propagation of microcracks within the soil. At this point, the rate of energy dissipation significantly accelerated, and the soil entered the macroscopic damage phase. Further analysis of the dynamic regulation effects of vibration frequency (as presented in Fig 13(D)–13(F)) revealed that higher frequency vibrations intensified interparticle collisions and friction, thereby promoting the rapid expansion of microcracks and a swift transition to macroscopic damage. Consequently, macroscopic experimental results demonstrated that, when vibration frequencies were set at 20 Hz, 35 Hz, and 50 Hz, increasing the vibration duration from 30 minutes to 90 minutes resulted in increments of disturbance degree *RD*_*q*_ by 0.06, 0.12, and 0.16, respectively, with the magnitude of increase progressively amplifying alongside higher vibration frequencies.

### Energy dissipation and damage evolution

According to the mechanical behavior and action mechanism of the elastic energy dissipation ratio illustrated in Fig 10. After being disturbed by vibrations, the structural integrity of the clay under unconfined compression deteriorates, accompanied by continuous energy dissipation during its damage evolution. Energy dissipation primarily includes frictional energy dissipation between particles, energy dissipation due to microcrack propagation, and plastic deformation energy dissipation. Initially, the pores in the sample are gradually compressed, leading to an increase in energy dissipation. Once the pores are completely closed, the soil structure becomes compact, and energy begins to accumulate in the form of elastic energy. As the load continues to be applied, microcracks keep expanding, causing a sharp rise in energy dissipation. If the external force persists, significant changes have occurred in its internal structure, and the mechanical behavior differs significantly from the initial pore closure stage. At this stage, new pore structures and contact patterns are formed within the soil, and the energy storage and dissipation mechanisms are reconstructed.

To our knowledge, research on structural and sensitive soils in this regard is currently scarce. We have sorted out the behavior and underlying mechanism of the elastic energy dissipation ratio obtained from the experiments, and attempted a comparative analysis with soils exhibiting structural and sensitivity characteristics. For instance, in expansive soils, the microstructure tends to be more compact in a dry state, potentially resulting in a lower energy dissipation ratio during the initial phase. As moisture infiltrates, the structure gradually loosens, leading to an increase in the energy dissipation ratio. This may significantly differ from the three-stage behavior typically exhibited by the elastic energy dissipation ratio presented in this study. Additionally, highly sensitive soft clay is prone to pronounced strain softening during shearing, characterized by a sharp drop in the dissipation ratio *K* after reaching the peak. This bears some resemblance to the stable trend observed in the third stage of the elastic energy dissipation ratio in this study, but the specific magnitude and rate of change vary depending on soil type. Through the above comparative analysis, we are able to further elucidate the mechanisms and influencing factors of elastic energy dissipation characteristics in different types of clay.

## Conclusion

This study investigates on the energy dissipation laws and underlying mechanisms of damage in Zhanjiang Formation structural clay following vibrational disturbances, by conducting unconfined compressive strength tests on disturbed samples with varying disturbance degrees. The stress-strain behavior of the damaged structural clay is analyzed, the compaction point of the samples is determined, and the energy evolution and distribution laws are explored. On this basis, the energy evolution mechanism is revealed. The key findings of the study are as follows:

1. The dissipation energy *W*^*Ω*^ of the damaged Zhanjiang Formation structural clay with varying disturbance degrees exhibits two distinct stages: slow growth and rapid growth. As the disturbance degree increases, the compaction strain also increases, indicating enhanced ductility of the samples. Concurrently, the compaction dissipation energy shows an increasing trend. Notably, samples with a higher disturbance degree possess more pores, thus requiring more dissipation energy to achieve densification.
2. Disturbances lead to a reduction in the strength of the Zhanjiang Formation structural clay damage samples; the increasing trend of dissipation energy at compaction points indicates that the deformation capability of the samples has been enhanced. The reason for this is that the combined effect of strength and deformation is a key factor influencing the amount of energy, and energy serves as the representation of the integrated impact of both strength and deformation.
3. The elastic energy dissipation ratio *K*, as a crucial parameter reflecting the proportional relationship between the energy storage and dissipation in soil, can effectively characterize the energy absorption and release capacity of the soil under stress. The soil exhibits different characteristics at various logarithmic stages of the elastic energy dissipation ratio *K*: In the initial stage of lg*K* > 0, it corresponds to the pore closure process in the soil. At this time, the pores between aggregates gradually decrease, the compactness of the soil gradually increases, and the stability continuously improves; when lg*K* < 0 to (lg*K*)_min_, the soil is in the elastic energy storage stage, and the total energy it absorbs is mainly stored as elastic energy. The proportion of elastic energy reaches its peak at (lg*K*)_min_, indicating the most stable state of the soil. From (lg*K*)_min_ to the failure stage, lg*K* commences to increase from the minimum value, signifying the transition of the soil from the elastic stage to the plastic stage and even failure; when lg*K* > 0 once again, it implies that the soil is approaching or has already failed. Thus, the (lg*K*)_min_ can be considered as the critical turning point for the transition from the elastic to the plastic stage of the soil. Furthermore, through Q-Q plot analysis, it was confirmed that the distribution of (lg*K*)_min_ conforms to the normal distribution, and its threshold distribution range was determined.
4. The dynamic regulation of damage energy in structural clay by vibration disturbance is mainly achieved by precisely controlling the vibration duration and frequency of the disturbance source. Short-term high-frequency disturbances may lead to rapid accumulation of pore water pressure within the soil, prompting redistribution of interparticle contact stress, thereby increasing instantaneous deformation and energy dissipation. Conversely, long-term low-frequency disturbances allow sufficient time for internal structural adjustments. Additionally, high-frequency vibrations may intensify friction and collisions between soil particles, fostering the formation of microcracks and accelerating cumulative damage.

## Supporting information

S1 Manuscript data. (ZIP)
